# Defining the extent of gene function using ROC curvature

**DOI:** 10.1093/bioinformatics/btac692

**Published:** 2022-10-22

**Authors:** Stephan Fischer, Jesse Gillis

**Affiliations:** Cold Spring Harbor Laboratory, Stanley Institute for Cognitive Genomics, Cold Spring Harbor, NY 11724, USA; Institut Pasteur, Université Paris Cité, Bioinformatics and Biostatistics Hub, Paris F-75015, France; Cold Spring Harbor Laboratory, Stanley Institute for Cognitive Genomics, Cold Spring Harbor, NY 11724, USA; Department of Physiology, University of Toronto, Toronto, ON, Canada

## Abstract

**Motivation:**

Interactions between proteins help us understand how genes are functionally related and how they contribute to phenotypes. Experiments provide imperfect ‘ground truth’ information about a small subset of potential interactions in a specific biological context, which can then be extended to the whole genome across different contexts, such as conditions, tissues or species, through machine learning methods. However, evaluating the performance of these methods remains a critical challenge. Here, we propose to evaluate the generalizability of gene characterizations through the shape of performance curves.

**Results:**

We identify Functional Equivalence Classes (FECs), subsets of annotated and unannotated genes that jointly drive performance, by assessing the presence of straight lines in ROC curves built from gene-centric prediction tasks, such as function or interaction predictions. FECs are widespread across data types and methods, they can be used to evaluate the extent and context-specificity of functional annotations in a data-driven manner. For example, FECs suggest that B cell markers can be decomposed into shared primary markers (10–50 genes), and tissue-specific secondary markers (100–500 genes). In addition, FECs suggest the existence of functional modules that span a wide range of the genome, with marker sets spanning at most 5% of the genome and data-driven extensions of Gene Ontology sets spanning up to 40% of the genome. Simple to assess visually and statistically, the identification of FECs in performance curves paves the way for novel functional characterization and increased robustness in the definition of functional gene sets.

**Availability and implementation:**

Code for analyses and figures is available at https://github.com/yexilein/pyroc.

**Supplementary information:**

Supplementary data are available at *Bioinformatics* online.

## 1 Introduction

Characterizing the functional properties of genes across conditions, species and other perturbations is a central challenge in post-genome biology. As datasets increase in size and complexity, exploiting methods from machine learning and AI research has become increasingly valuable to parse vast data collections for subtle convergent signals (Berger *et al.*, 2013; Le, 2020; Libbrecht and Noble, 2015; Mahood *et al.*, 2020; Mostafavi *et al.*, 2008). However, the complexity and variety in formalism of these methods create interpretation problems of their own. Establishing a consensus framework to evaluate prediction accuracy and identify features driving prediction accuracy has been essential to progress, often using systematic data resources, and with well-defined performance metrics. In particular, many problems in genomics map to a supervised learning framework with a goal of determining functional sets of genes from partial annotations and feature data. A correspondingly high number of methods and assessments report comparative evaluation using traditional machine learning statistics, such as the area under the receiver-operator characteristic curve (AUROC). However, genomics poses unique challenges and opportunities relating to the extreme scalability of data collection and analyses, both across novel contexts, such as conditions, tissues or species, and the ability to collect high-throughput data in consistent assays.

The shared ancestry of organisms forms the basis of many ways we extend results from one system to another. Across species, this shared ancestry is the basis for functional annotation using homology (Altenhoff *et al.*, 2015); within species, it is the basis for a shared reference to align functional genomics data (Eraslan *et al.*, 2019; Mostafavi *et al.*, 2008). Both of these foundational ideas exploit the shared existence of the same set of genes across systems, placing data collected from heterogeneous sources into a common framework. Whenever a gene is described as linked to a disease (Le, 2020; Wong *et al.*, 2021), annotated with a Gene Ontology (GO) function (Ashburner *et al.*, 2000; The Gene Ontology Consortium, 2021) or described with respect to structure or biochemical activity (Capra and Singh, 2007; Lee *et al.*, 2007), we imply a standardized description of the ‘same’ gene found in different systems. Analytically, this frequently creates an oddity within machine learning of gene function: because samples are genes, we are learning over the same sample space, again and again, extending an initial positive set to include more and more of what were originally negatives (Le, 2020). This is unlike supervised learning in any other field where the intent is to learn a classifier that can be applied to ‘new’ samples, as opposed to the same samples over again. As a result, generalizability can only be assessed across systems, such as conditions, rather than samples; i.e. we ask does this new experiment also imply a gene possesses a given function? Combined with using primarily sparse positive annotations without explicit negatives (Dessimoz *et al.*, 2013; Thomas *et al.*, 2012; Youngs *et al.*, 2014), this separate ‘closed universe’ problem of resampling across novel feature spaces makes it difficult to interpret annotation performance from traditional machine learning metrics alone.

A second challenge relates to the magnitude of genome-scale data. In modern genomics, many assays are designed to be comprehensive across the genome, with significance arising from the combination of information across genes. This is used in differential expression (de la Fuente, 2010), enrichment analysis (Irizarry *et al.*, 2009; Subramanian *et al.*, 2005) and more generally, network analyses that aim to capture gene associations of all types (Barabási and Oltvai, 2004; Mostafavi *et al.*, 2008). Thus, networks can be interrogated for overlaps in disease genes or other sets, with even a small number of genes contributing to generating a significant result if they are ‘surprisingly’ close in the network. More broadly, there are two potentially complementary models for gene associations: in the first model, functions and phenotypes are well captured by a small set of genes [Mendelian diseases or large effect loci in GWAS (Park *et al.*, 2011; Gibson, 2012)], while in the second model functions are distributed over a large set of genes [polygenic model (Gibson, 2012; Golan *et al.*, 2014), omnigenic model (Boyle *et al.*, 2017)]. In both models, proteins frequently participate in multiple functions, resulting in overlap between gene sets (Crow *et al.*, 2019; Gillis and Pavlidis, 2011a), reflecting poor human definitions for functions or true multifunctionality. Likewise, diffuse interactions may reflect noisy data or true omnigenic robustness (Mihalik and Csermely, 2011). To understand these questions about the discreteness and extent of gene function, we need a framework that lets us interpret conclusions drawn in one context jointly with others.

In this article, we assess the generalizability of gene associations based on the graphical properties of performance curves. We start by showing that genomic ROC curves endemically produce highly significant straight segments across a selection of 50 articles covering a wide body of methods and data. Using a toy model and simulated data, we illustrate how each straight segment groups together annotated and unannotated genes that are equally likely to have the investigated function. We show that the presence of straight lines can be assessed using the normalized Kolmogorov–Smirnov statistic. Systematizing our observations from published curves, we show that straight lines are pervasive across data sources and gene functions, suggesting the existence of large gene modules (up to 40% of the genome). Finally, we show how straight lines in ROC curves enable us to rapidly evaluate the generalizability of gene sets across virtually any study, and they can be used to tailor pre-existing gene sets to a new context. Together, these results and methods for the interpretation of performance curves extend our ability to rapidly and visually probe gene set generalizability across studies and systems.

## 2 Materials and methods

In the following, we considered three sources of ROC curves and designed several metrics to characterize the shape of these ROC curves.

### 2.1 ROC curves from the literature

We systematically sampled 35 ROC-curve-containing research articles from the *PLoS One* journal during one calendar year (genomics-related Subject Areas) and selected 15 high-profile research articles (see Supplementary Appendix S1 for a detailed list of papers and figures extracted). We used the Engauge Digitizer (https://doi.org/10.5281/zenodo.3941227) software to extract curves from the selected figures, following the standard procedure (selection of 3 axis points for scale, automatic segment detection). In instances where the figure contained too many overlapping curves and individual curves proved too difficult to extract, we removed the figure from the analysis. After the extraction process, Engauge Digitizer generated CSV files with data points evenly distributed along the curve. To harmonize the curve resolution, we interpolated the curves such that they contained 200 total points evenly spaced along the *x*-axis (FPR axis).

### 2.2 ROC curves from PPI, co-expression and co-domain data

We downloaded the mouse PPI data BIOGRID-ALL version 4.4.197 from the BIOGRID website (Oughtred *et al.*, 2019; Stark *et al.*, 2006). We filtered the BIOGRID data for mouse (taxonomy ID 10090) and physical interaction (‘Experimental.System.Type’ == ‘physical’). This initial network contained 57 337 interactions across 10 172 genes. To take into account indirect connections (Gillis and Pavlidis, 2011b), we propagated the existing interactions, setting the weight for each pair of proteins as the inverse of the shortest path between the two proteins.

We downloaded the mouse co-expression network from the CoCoCoNet (Lee *et al.*, 2020) website (last updated on April 20, 2021). We converted Ensembl identifiers gene to symbols using the mapIds function from the AnnotationDbi and the org. Mm.eg.db R packages (last updated on April 21, 2021), resulting in a dense network of 17 834 genes. Finally, we subset the co-expression and PPI networks to common genes, resulting in networks of 9058 genes.

We downloaded protein domain information from UniprotKB (The UniProt Consortium, 2021) using the REST API. For each mouse protein, we extracted domain information from the ‘Domain [FT]’ column, obtaining a one-hot encoded matrix for 7269 proteins and 2161 domains. We computed a protein–protein similarity matrix by normalizing the one-hot matrix using the TF-IDF transformation, then taking the cosine similarity. Finally, we propagated similarities using the same shortest path algorithm as used previously for the PPI data.

We downloaded the Gene Ontology (GO) (Ashburner *et al.*, 2000; The Gene Ontology Consortium, 2021) from the GO website (GO-basic table in OBO format, last updated December 18, 2019). We downloaded gene ontology annotations from the MGI website (‘gene-association’ table, last updated September 9, 2019) and automatically propagated annotations to higher level terms. For downstream analyses, we only kept 4238 GO terms with ≥20 annotated genes after restricting to the 9058 common genes.

We computed ROC curves by assessing whether GO terms are preferentially connected in the three networks using EGAD (Ballouz *et al.*, 2017). We re-implemented EGAD’s modularity metric in Python. Following the original algorithm, we implemented 3-fold Cross-Validation (CV), with 2/3 of positives used for training, and 1/3 of positives held-out for testing. In detail, let $X_{ij}$ be a positive symmetric adjacency matrix representing a weighted network, where i and j range from 1 to *N* (number of genes). Let $P_{i}$ be the one-hot encoding of the training positives. The EGAD algorithm was reproduced by computing the node degree $D_{i}=\sum_{j}X_{ij}$, the neighbor votes V=X.P and the normalized neighbor vote $V_{i}^{\prime }=V_{i\;}/{\;D}_{i}$. The normalized neighbor votes were used as a predictor of held-out positives, yielding one ROC curve per CV fold. For a given gene set, the final ROC curve is reported as the average ROC curve (across the 3 CV folds), the final AUROC as the average AUROC.

We downloaded drug-target interaction information from STITCH v5.0 (Szklarczyk *et al.*, 2016) from the EMBL website. We extracted the top 10% protein–target interactions according to the ‘combined_score’ column, resulting in a binary matrix with 14 190 proteins and 449 815 drugs. For downstream analyses, we only kept drugs with interactions with more than 20 proteins. We computed ROC curves using EGAD as described above, except using drugs as labels instead of GO terms.

### 2.3 ROC curves from single-cell RNA sequencing data

We downloaded the Tabula Muris (Schaum *et al.*, 2018) single-cell RNA sequencing (scRNAseq) dataset from FigShare, specifically Version 2 of the 10× [Single-cell RNA-seq data from microfluidic emulsion (v2), 2018] and Smart-Seq2 [Single-cell RNA-seq data from Smart-seq2 sequencing of FACS sorted cells (v2), 2018] data, along with metadata and annotations, keeping all annotated cells (100 605 cells). We applied CP10K (counts per 10k) normalization for the 10× data and CPM (counts per million) normalization for the SmartSeq data.

We computed markers for each mouse by tissue combination using the MetaMarkers package (Fischer and Gillis, 2021). We subset the datasets to a given mouse using the ‘mouse_id’ metadata, then ran the compute_markers function on the normalized counts, using the ‘cell_ontology_class’ as cell type labels, and ‘tissue’ metadata as group labels (stratifying marker search by tissue). We removed genes with low detection rate (<10% in all mice by tissue by cell type combinations) and only kept markers inferred for cell types containing at least 20 cells.

To compute ROC curves, we asked if a set of reference markers were the top markers in other mouse by tissue combinations. For each mouse by tissue combination, we ordered genes according to the effect size of the ROC test, ‘auroc’ column in the MetaMarkers table, then used this list as a predictor for the reference markers. We used the ‘prediction’ and ‘performance’ functions from the R ROCR package to compute the ROC curve (‘tpr’ and ‘fpr’ statistics) and the AUROC (‘auc’ statistic).

We obtained a first set of reference markers by selecting the top 20 markers for the ‘3_10_M’ mouse from the Smart-Seq dataset, for the ‘B cell’ cell type in the ‘Fat’ tissue. To determine the second set of reference markers, we visually estimated that the top 2 FECs in ‘Lung’ spanned 5% of negatives. We extracted and pooled the top 5% markers (ranked by MetaMarkers ‘auroc’) in all 4 individuals (‘3_39_F’, ‘3-F-57’, ‘3-F-56’, ‘3-M-7/8’), resulting in a marker set of 480 genes. To determine the third set of reference markers, we visually estimated that the top 2 FECs in ‘Spleen’ spanned 1% of negatives. We extracted and pooled the top 1% markers in all 8 individuals (‘3-M-8’, ‘3-F-56’, ‘3_8_M’, ‘3_9_M’, ‘3_11_M’, ‘3_10_M’, ‘3_38_F’, ‘3_39_F’), resulting in a marker set of 216 genes.

### 2.4 Simulated ROC curves

To investigate how straight segments arise in ROC curves, we designed two simulation models with controlled modularity. We refer to these models as the Gaussian model and the network-based model.

In the Gaussian model, we consider an ensemble of 10 000 genes. When a gene’s functionality is assessed, it obtains a score that follows a N(μ,σ) Gaussian distribution. The parameter μ reflects the gene’s functional state. In the simple ‘on/off’ model, μ=0 if the gene is non-functional, μ=1 if the gene is functional; 20% of genes are labeled as functional. We also consider a ‘on/low/off’ model (μ∈{0,0.5,1}, mixing proportions {0.5, 0.3, 0.2}) and a ‘continuous’ model with 4 states (μ∈{0,1/3,2/3,1}, mixing proportions {0.4,0.3,0.2,0.1}). We simulate two assessments. The first assessment is used to annotate genes: it is simulated under a given noise level σ (σ = 1/2, 1/3 and 1/4 for the ‘on/off’, ‘on/low/off’ and ‘continuous’ models, respectively), all genes that exceed a score of 0.2 are considered functional and annotated as positives. The second assessment independently re-evaluates the gene’s functionalities at increasing levels of noise (σ∈{0.1,0.2,0.3,0.4}) and compares them with the initial annotation.

In the network-based model, we consider a network of 10 000 genes composed of 4 pre-defined communities of 2500 genes each. For the initial annotation assessment, each gene’s label is set according to an annotation probability P that depends on the functional state of each community. We consider four models: non-modular function (P=0.5 for all communities), on/off function (P=0.8 for the functional community, P=0.1 otherwise), on/low/off function (P∈{0.8, 0.5,0.1}) and a continuous function (P∈{0.4,0.3,0.2,0.1}). Once the labels have been drawn, the modularity of annotated modules is re-evaluated using the EGAD algorithm (see Section 2.2) under varying levels of observed modularity. Starting from the pre-defined communities, the observed modularity follows the following block structure: any two nodes from different communities are connected with a weight following a N(2,2) distribution, while two nodes from the same community are connected with a weight following a N(μ,2) distribution where μ∈{2,2.1,2.2,2.3) (increasing observed modularity).

### 2.5 Assessment of linearity using the Kolmogorov–Smirnov statistic

Conceptually, the one-sample Kolmogorov–Smirnov (KS) statistic measures the maximal deviation of a Brownian bridge, a random walk with fixed starting and ending points. Under random labeling of positives and negatives, the ROC curve can be seen as a random walk in (TPR, FPR) space (Fig. 2); the ‘randomness’ of annotation (local equivalence of positives and negatives) can thus be evaluated from the KS statistic.

Formally, we assessed the linearity of an ROC subcurve by rescaling it to a [0,1] by [0,1] square, then computing the deviation from the diagonal line. Mathematically, given a subcurve starting at the ($\mathrm{FP}R_{0}$,$\mathrm{TP}R_{0}$) point and ending at the ($\mathrm{FP}R_{1}$,$\mathrm{TP}R_{1}$) point, the rescaled subcurve is given by $\mathrm{FP}R^{'}=\;\left(\mathrm{FPR}-\mathrm{FP}R_{0}\right)/(\mathrm{FP}R_{1}-\mathrm{FP}R_{0})$ and $\mathrm{TP}R^{'}=\left(\mathrm{TPR}-\mathrm{TP}R_{0}\right)/(\mathrm{TP}R_{1}-\mathrm{TP}R_{0})$. The deviation from the diagonal (KS statistic) is $D_{n}=\sup\left(\left|\mathrm{TP}R^{'}-\mathrm{FP}R^{'}\right|\right)$ and the normalized KS statistic is $D^{\prime }=D_{n}.\sqrt{n}$, where *n* is the number of positives. To compute *P*-values, we used the C_pKS2 function used by the R function ks.test, corresponding to a one-sample test with uniform distribution and parameter ‘exact=FALSE’.

For ROC curves computed from PPI data, co-expression data, co-domain data and curves extracted from articles, we automatically identified the longest linear segments. We considered all possible subcurves (start/end point combinations) spanning at least 5% negatives ($\mathrm{FP}R_{1}-\mathrm{FP}R_{0}\geq 0.05)$, then computed the normalized KS statistic D′ as described above. For articles, the number of positives was generally unknown and was set to *n* = 100. We tagged all subcurves with D′≤1 (asymptotic *P*-value of *P* ≥ 0.27) as linear, then established the final list of FECs by iteratively extracting the longest non-overlapping straight lines. For ROC curves computed from single-cell data, we visually assessed the extent of the initial FEC segments, then confirmed their significance using the KS test.

### 2.6 Longest segment approximation of ROC curves

For each ROC curve, we extract the longest linear segment as described above, then reduced the ROC curve to 4 points: (0,0), ($\mathrm{FP}R_{0}$,$\mathrm{TP}R_{0}$), ($\mathrm{FP}R_{1}$,$\mathrm{TP}R_{1}$), (1,1), where ($\mathrm{FP}R_{0}$,$\mathrm{TP}R_{0}$) and ($\mathrm{FP}R_{1}$,$\mathrm{TP}R_{1}$) are the two extremities of the longest linear segment. We then computed the AUROC using the trapezoidal rule.

### 2.7 Extraction of optimal ROC subcurve flip

For each ROC curve, we recomputed the AUROC after flipping each possible subcurve using the trapezoidal rule, then identified the subcurve flip that resulted in the highest AUROC.

## 3 Results

### 3.1 Straight lines in ROC curves are pervasive in the genomics literature

**Fig. 1.**
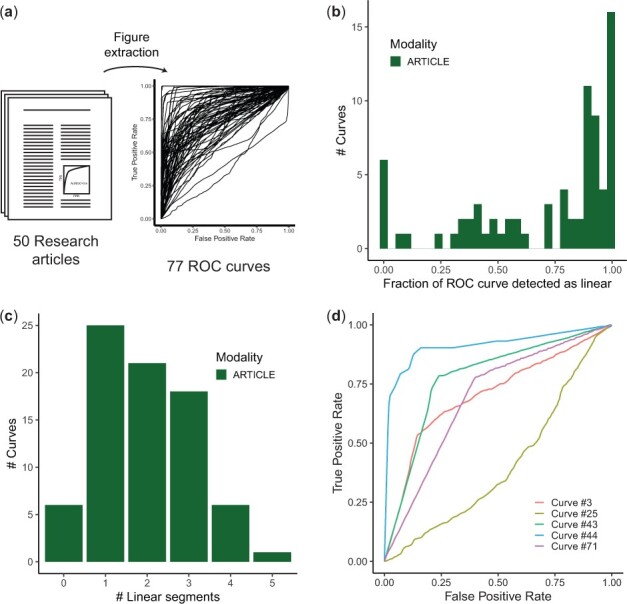
Straight segments in ROC curves are surprisingly common in research articles. (**a**) ROC curves were extracted using the Engauge Digitizer tool from 15 selected publications and 35 publications published during one calendar year in genomics-related subject areas of the *PLoS One* journal. (**b**) Fraction of ROC curve detected to be straight lines (Kolmogorov–Smirnov test). (**c**) Number of linear segments per curve. (**d**) Examples of ROC curves composed almost entirely of straight lines

While reviewing the genomics literature, we were struck by the recurrence of straight lines in published ROC curves. To confirm our intuition that straight segments are surprisingly common, we extracted ROC curves from 50 research articles, composed of an unbiased selection of 35 articles from the *PLoS One* journal and 15 manually curated high-profile articles (Supplementary Appendix S1). In total, we extracted 77 ROC curves using the Engauge Digitizer (see Section 2) software (Fig. 1a).

The predictions summarized by the ROC curves were either gene-centric predictions (74/77 curves) or interaction predictions (3/77 curves). From the methods description and the figures, we estimated that the number of positives ranged from 24 to 6000, while the total number of objects ranged from 121 to 119 149. In most cases, the prediction problem was heavily class imbalanced, with positives representing 1–10% of objects, but we identified at least 4 curves with matching numbers of positives and negatives. Overall, the extracted curves sample a wide variety of learning problems as they are typically formulated in the genomics field.

**Fig. 2.**
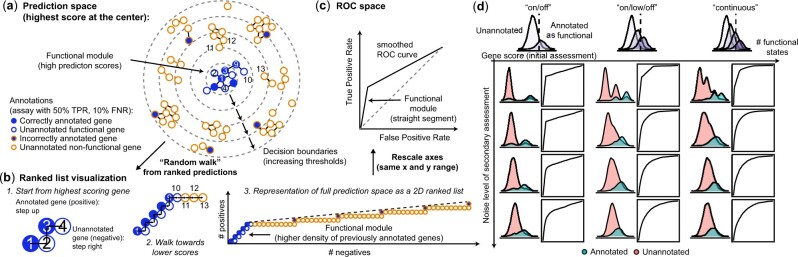
Straight lines in ROC space reveal the presence of functional modules. (**a**) Schematic representation of a gene function prediction task. Nodes represent genes, edges represent the strength of interactions between genes as determined by a high-throughput assay (for simplicity, only strong interactions are shown). The fill color of nodes shows the genes’ current annotation (as established by a low-throughput assay), the outline color shows the true status, numbers show the genes’ prediction rank. Genes are organized according to prediction scores from a machine learning classifier, with genes most likely to be functional (highest predictions scores) at the center. (**b**) Taking current annotations as the ground truth (positive = annotated, negative = unannotated, closed-world assumption), predictions can be summarized as an ROC curve. The ROC curve can be conceptualized as a walk in (FPR, TPR) space: starting from the gene with the highest prediction score, the curve moves up every time an annotated gene is encountered, and right every time an unannotated gene is encountered. (**c**) The presence of a functional module that mixes annotated and previously unannotated gene is revealed by the presence of straight lines in the ROC curve. (**d**) ROC curves built from a simulation model where gene scores follow a Gaussian mixture model. Gene functionality is assessed twice. An initial noisy assessment annotates all genes that exceed a certain score as functional, a second independent assessment re-evaluates the gene functionality and computes the agreement with the initial annotations as an ROC curve

We assessed the presence of straight lines using the normalized Kolmogorov–Smirnov statistic (Section 2) and found that 92% (71/77) curves contained straight segments, spanning 71% of the curve on average (Fig. 2b). 31/77 curves were composed almost entirely of straight lines (covering >90% of the curve), with 39/77 curves contained exactly 2 or 3 segments (Fig. 2c and d). In summary, straight segments are extraordinarily recurrent in the genomics literature and a surprising number of curves are piecewise-linear.

### 3.2 Straight lines in ROC space suggest the presence of discrete functional modules

Why are straight lines so common in genomics ROC curves? To provide an initial intuition, we consider a toy example of protein function prediction (Fig. 2). A machine learning classifier is applied to a high-throughput dataset measuring the likelihood that two proteins interact (Fig. 2a). The classifier is trained on sparse annotations obtained by a low-throughput assay that labeled a subset of genes with 50% True Positive Rate (TPR) and 10% False Negative Rate (FNR) (Fig. 1a). In this ideal scenario, the classifier identifies a functional module containing an even mix of previously annotated genes and unannotated functional genes.

The presence of this functional module in the data is immediately visible on the ROC curve through the presence of straight lines. This can be intuitively appreciated when visualizing the gene scores as a two-dimensional ranked list (Fig. 2b). Compared to a simple one-dimensional ranking of genes, the annotation labels are also represented by organizing genes according to a ‘random’ walk: if a gene was previously annotated, the walk steps up, otherwise, the walk steps right. This representation makes it visually obvious that, in the predictions, there is a group of genes that contains a high density of previously annotated gene in the form of a quasi-straight segment at the beginning of the walk (Fig. 2b). Up to the rescaling of axes, this 2D ranked list representation is identical to the ROC curve (Fig. 2c).

To further illustrate when we expect to find straight lines along the ROC curve, we consider a simple simulation model of discrete groups of genes with varying degrees of functionality (e.g. not functional, lowly functional, strongly functional, Fig. 2d). In an initial assessment, all the genes that pass a certain score are annotated as functional. Since the assessment is noisy, some functional genes are missed and some non-functional genes are incorrectly annotated. In a second independent assessment, the genes’ functionality is re-evaluated and compared against the initial annotation in the form of an ROC curve. The aspect of the curve critically depends on the quality of the second assessment: at low levels of noise, straight lines in the ROC curves reveal the number and extent of functional states, while at higher levels of noise, the ROC becomes curved, hiding the latent discrete nature of the data. We obtained similar conclusions for simulations of a network model with varying degrees of modularity and guilt-by-association predictions (Supplementary Fig. S1).

In summary, the presence of a straight segment in ROC space suggests: (i) the existence of a discrete module of genes with high prediction scores, (ii) a potential mismatch between existing annotations and the module suggested by the predictions, (iii) the equivalence or interchangeability of genes within the module, which causes previously annotated genes and unannotated genes to be evenly mixed. As a result, the initial straight line on the ROC curve suggests that the annotation can be ‘naturally’ extended to some unannotated genes as, locally, annotated and unannotated genes are equivalent.

To emphasize the interchangeability of annotated and unannotated genes, we refer to straight lines as *Functional Equivalence Classes* (FECs). Note that we are specifically interested in straight lines in the absence of ties, which arise when the score distribution of a subset of negatives and positives is identical (Fig. 2d), i.e. the class labels are locally interchangeable (Supplementary Fig. S2). The permutability of class labels can be assessed with the normalized Kolmogorov–Smirnov statistic, allowing us to automatically detect straight lines in any ROC curve (see Section 2).

### 3.3 FECs are pervasive across the functional landscape

The common occurrence of FECs in the published literature may be explained in the light of network biology, which identifies fundamental functional building blocks by analyzing the global topology of molecular interaction networks (Barabási and Oltvai, 2004). The central hypothesis is that there are robust building blocks whose interactions are shaped by evolution. This hypothesis serves as the foundation of widespread applications such as gene set enrichment analyses (Irizarry *et al.*, 2009; Subramanian *et al.*, 2005), which look for functional enrichment across a pre-defined hierarchy of discrete gene sets [such as the Gene Ontology (Ashburner *et al.*, 2000; The Gene Ontology Consortium, 2021) or MSigDB (Subramanian *et al.*, 2005)]. However, it remains difficult to test how well discrete gene sets are supported by the data, and how context-dependent they are.

To assess the presence and extent of discrete modules across the functional landscape, we turned to a broad set of functions as defined by the GO and investigated the presence of FECs across two types of network data offering wide meta-analytic resources and capturing different aspects of function: Protein–protein interaction (PPI) networks and co-expression networks. PPI networks are binary networks where nodes are proteins and edges connect pairs of proteins that physically interact. In contrast, co-expression networks are weighted networks where nodes are genes and edges reflect the propensity of two genes to be expressed in the same contexts (conditions, tissues or cell types).

We built a PPI network by aggregating all interactions from the BIOGRID (Oughtred *et al.*, 2019; Stark *et al.*, 2006) database annotated as ‘Mouse’ and ‘Physical Interaction’, resulting in a network containing 10 172 proteins and 57 337 interactions. As PPI networks are typically sparse, we used a propagation algorithm to obtain a dense network, which accounts for indirect interactions between proteins (see Section 2). We downloaded the mouse co-expression network from the CoCoCoNet (Lee *et al.*, 2020) database. The network was obtained by aggregating 3359 samples over 85 experiments, resulting in a dense network containing 17 834 genes. To allow comparisons between the two modalities, we restricted the two networks to 9058 common genes.

**Fig. 3.**
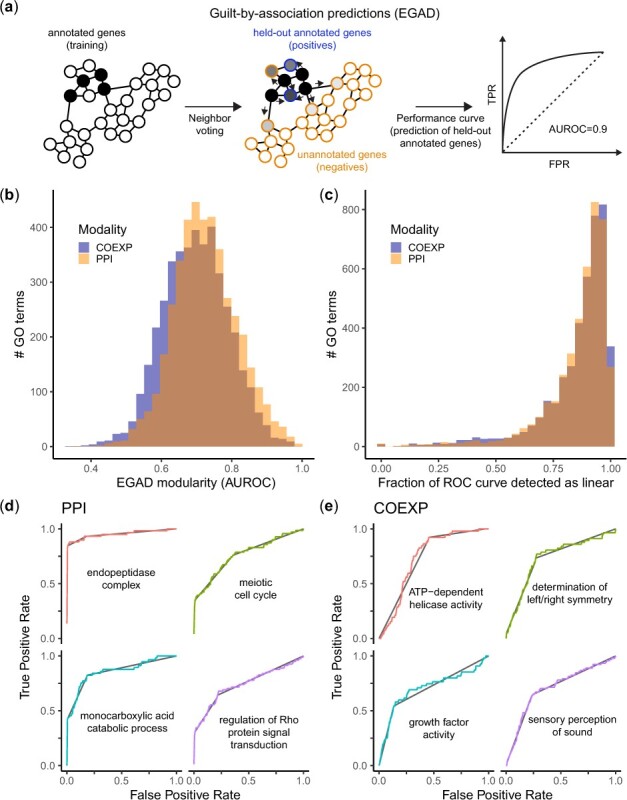
FECs are pervasive across the functional landscape. (**a**) Schematic of the function prediction task. EGAD predicts functional annotations based on the connectivity of genes in network data (neighbor voting algorithm). Using held-out annotated genes as positives, performance can be summarized as an ROC curve, which reflects the degree of modularity of a functional gene set. (**b**) Degree of modularity (EGAD AUROC) of functional gene sets defined by the Gene Ontology (GO) in meta-analytic co-expression (COEXP) and PPI data. (**c**) Fraction of ROC curves detected to be straight lines. (**d**, **e**) Examples of ROC curves composed almost exclusively of straight lines. Each facet shows a specific GO term, colored curves show the ROC curve for this term, black lines show the FECs detected using the KS test

To assess whether a function is supported by a network’s topology, we used the guilt-by-association framework implemented by the EGAD (Ballouz *et al.*, 2017) algorithm. Briefly, EGAD uses a neighbor voting algorithm to assess whether genes that are annotated with the same function tend to be neighbors in the network (Fig. 3a). Some of the annotated genes are held-out and serve as positives, while all other genes are annotated as negatives [closed world assumption, (Dessimoz *et al.*, 2013)]. Taking neighbor votes as a predictor for held-out genes, we build one ROC curve for each function and network. A high AUROC indicates that the annotations are supported by the network, i.e. genes annotated with this function tend to belong to the same module. Overall, GO functions were strongly supported by both the PPI (median AUROC = 0.72) and co-expression networks (median AUROC = 0.70, Fig. 3b). Performance was only partially correlated (rho = 0.35, Supplementary Fig. S3), consistent with the fact that PPI and co-expression capture different aspects of function.

Straight segments were extremely common in both types of data, suggesting widespread modular structure across the genome. FECs that spanned at least 5% of the genome were detected in 99.8% functions and spanned 85% of the genome on average (Fig. 3c). 95/8478 (1.1%) functions were even detected to be entirely composed of straight lines, such as ‘meiotic cell cycle’ (2 FECs, Fig. 3d) or ‘determination of left/right symmetry’ (2 FECs, Fig. 3e).

**Fig. 4.**
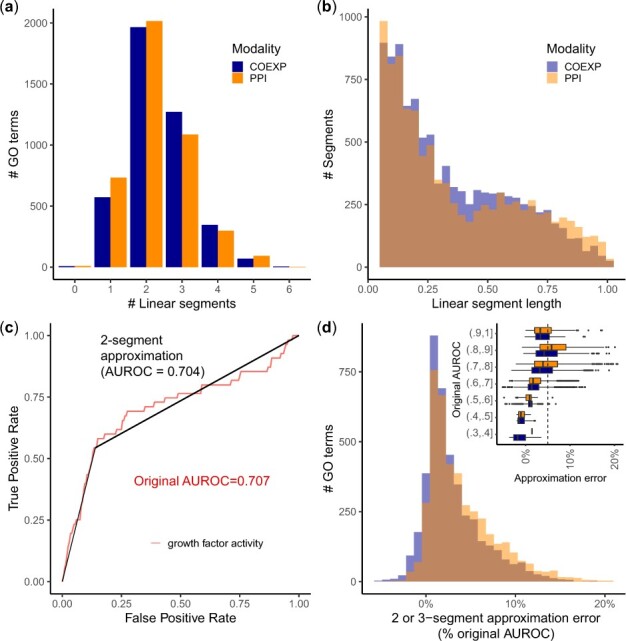
FECs offer a data-driven view of the extent of biological functions. (**a**) Distribution of the number of FECs detected in ROC curves corresponding to each GO term. (**b**) Distribution of length of individual FECs, as measured by the fraction of the *x*-axis (FPR axis) spanned by the FEC. (**c**) Example of 2-segment approximation for the ‘growth factor activity’ GO term. The initial ROC curve and AUROC are shown in red, the straight line approximation and approximated AUROC are shown in black. (**d**) Distribution of approximation error on the AUROC when swapping ROC curves by their 2 or 3-segment approximation. A low approximation error suggests that performance is driven by the presence of 2 or 3 discrete modules in the data. The inset further breaks down the approximation error by stratifying on the original AUROC

3980/8476 functions (46% in co-expression, 48% in PPI) contained exactly two FECs (Fig. 4a), suggesting a binary partition of the genome (function-associated versus non-functional). The length of individual FECs varied substantially across functions and had a clear bimodal shape in both modalities (Fig. 4b). The first mode contained 62% of FECs and spanned 5% to 40% of the genome; it roughly corresponded to the length of the primary FEC of each curve, i.e. the FEC containing the highest-ranking genes (Supplementary Fig. S4).

Because FECs often spanned large portions of the genome and most curves contained exactly two or three FECs, we wondered how many functions could be explained by the presence of two or three discrete classes of genes, corresponding to the ‘on/off’ and ‘on/low/off’ simulation models. We identified the start and end of the longest FEC in each curve, replaced it by a straight line, then connected this line to the (0,0) and (1,1) points using straight segments. In cases where the FEC contained the (0,0) or (1,1) point, the ROC curve was approximated by two straight lines (Fig. 4c). We found that the two or three line-approximation worked to a surprising degree: 74% of curves could be approximated with a relative error on the AUROC lower than 5% (Fig. 4d).

Despite partially uncorrelated performance, the presence and size of FECs was remarkably consistent across the PPI and co-expression modalities. We show that these distributions hold across an even larger body of data, including the ROC curves from the literature (Supplementary Fig. S5), protein function prediction from protein domain information (Supplementary Fig. S6), and drug–target interaction predictions (Supplementary Fig. S7). Overall, these results suggest that modular structure is widespread in the data, although modules only partially overlap with existing annotations. For most functions, the data even suggest a binary partition of genes, with function-associated genes constituting up to 40% of the genome.

### 3.4 FECs capture context-dependent views of functionality

While the presence of FECs suggests modular structure in the data, the slope and extent of FECs suggest that data-driven modules only partially overlap with existing annotations. This shift in annotation may arise from imperfect annotations, but it may also reflect the fact that the same function is associated with different gene sets depending on the context.

To obtain context-specific functional gene sets, we turned to cell-type-defining genes identified from single-cell RNA sequencing (scRNAseq) data. Recent cell type atlasing efforts suggest that mature cell types act as discrete transcriptomic entities and constitute conserved building blocks of biology (Bakken *et al.*, 2020; Baron *et al.*, 2016). In transcriptomic space, this discrete nature translates into well-separated clusters and cell-type-specific marker genes.

To evaluate the replicability of marker modules across contexts, we extracted markers from the Tabula Muris atlas (Schaum *et al.*, 2018), which contains 100 605 cells sampled from 7 mice (3 males and 4 females) across 20 organs. We focused on the ‘B-cell’ cell type, detected in 42 combinations of individuals (7 individuals), tissues (7 organs) and sequencing technologies (10X and Smart-Seq) for a total of 10 323 cells.

We extracted the top 20 cell type markers (see Section 2) from the ‘3_10_M’ individual in the ‘Fat’ tissue, sequenced using the Smart-Seq technology. This corresponds to a typical marker gene extraction scenario, in which a study relies on a single tissue and sequencing technology. To study the generalizability of these 20 markers, we asked whether they are also predicted as top markers in the remaining data. We generated one ROC curve per individual, tissue and technology combination that contained more than 20 cells (25 combinations).

**Fig. 5.**
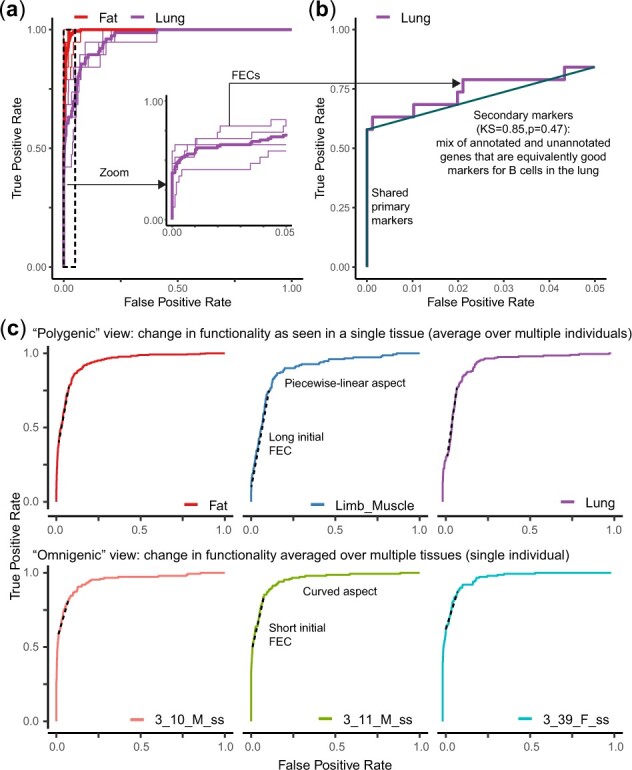
Straight lines identify generalizable markers and tissue-specific markers. (**a**) Evaluation of the generalizability of the top 20 fat markers obtained from a single individual. Thin lines show individual ROC curves (one mouse, tissue, technology combination), thick lines show average ROC curves per tissue. A high AUROC indicates that markers generalize well, i.e. they are also top markers in other tissue *x* technology combinations. Inset: a zoom on the ROC curve up to 5% FPR highlights the presence of straight lines in the Lung. (**b**) The presence of straight lines can be assessed using the Kolmogorov–Smirnov (KS) test. The initial straight lines can be used to identify primary markers (perfect markers shared across tissues) and secondary markers (tissue-specific markers). Secondary markers contain a mix of previously annotated genes and unannotated genes with equal marker strength. (**c**) Same assessment as (a), but taking primary and secondary markers from the Lung as the reference marker set. Inset: a zoom on the ROC curve up to 1% FPR highlights the presence of straight lines in the Spleen. (**d**) Same assessment as (a), but taking primary and secondary markers from the Spleen as the reference set

Marker replicability AUROCs ranged from 0.83 to 1 (median 0.97), suggesting high overall replicability. Performance differences were mostly explained by variability across tissues (61% variance explained). Compared to markers extracted from the same tissue, but from different individuals, the markers displayed perfect replicability (AUROC ∼ 1, lines labeled “Fat” in Fig. 5a). However, performance in other tissues was suboptimal (AUROC < 1, lines labeled “Lung” in Fig. 5a). In the initial portion of the ROC curves, we identified two straight lines spanning approximately 5% of the genome (Fig. 5a). The first FEC (perfect straight line, KS = NA) highlighted that approximately 50% (∼10/20) markers picked in fat were perfectly replicable in lung (Fig. 5b). We call these genes primary markers. In contrast, the second FEC (KS = 0.85, *P* = 0.47, *n* = 5) contained approximately 25% of the original markers, but also 5% of the negative genes with equivalent marker strength. This line suggests the existence of context-dependent markers: markers extracted from fat can be completed with previously unidentified genes that can be used interchangeably as secondary markers in the lung. Lung-specific secondary markers were consistent across all individuals and technologies, suggesting that they fundamentally reflect differences across tissues (Supplementary Fig. S8a). As a result, we can automatically extend the starting marker set to a larger and more robust set of markers. The first two FECs represent a new candidate set with 480 markers (16/20 initial genes, 464 additional genes) which are highly replicable across tissues (AUROC > 0.96, ΔAUROC=-0.03–0.03), with the notable exception of the mammary gland (ΔAUROC = –0.09) and the spleen (ΔAUROC = −0.36, Supplementary Fig. S8b).

This procedure can be repeated: FECs can be used to adapt any pre-existing gene set to a different context. Building an ROC curve for the spleen against the 480 lung markers, the two initial FECs (KS = 1.2, *P* = 0.12, *n* = 18 for the average ROC curve, Supplementary Fig. S8c) contain ∼25% of the lung marker set and 1% negative genes with equivalent performance in the spleen, representing a total of 216 genes. This new marker set resulted in perfect performance in the spleen and lower performance in other tissues (AUROC range = 0.89–0.95, Supplementary Fig. S8b), suggesting that most of the newly identified markers are spleen-specific.

Remarkably, when ROC curves are used to evaluate the generalizability of a marker set from a context A to a context B, they tend to contain long initial FECs, suggesting the presence of marker modules that shift across contexts, but remain discrete modules in any given context (Fig. 5c). However, when ROC curves are averaged across multiple contexts, the ROC curve becomes heavily non-linear (Fig. 5c). The non-linearity suggests the existence of a functional gradient, reflecting the fact that some genes tend to be associated with B cell functionality more frequently when a wide variety of contexts are considered.

This example shows how FECs decompose a candidate gene set into discrete classes of genes with respect to a given functional property. A simple look at a set of ROC curves suggests the existence of shared (primary markers) and tissue-specific properties (secondary markers). The size of straight lines can be directly interpreted: there are around 10–50 primary markers and 100–500 secondary markers. The presence of FECs seems to directly depend on the assessment: when a single context (tissue) is considered, the gene organization is modular, across multiple contexts, there is a hierarchy of genes that tend to be more frequently associated with the function.

## 4 Discussion

In this study, we showed that the shape of ROC curves offers a visual and data-driven interpretation of the extent of biological functions. The presence of straight lines in the ROC curve suggests that the data are compatible with the extension of a functional gene set to unannotated genes. We call these straight lines FECs, because they define discrete classes of genes that are equivalent with respect to the functional property investigated. Our examples show that the extensibility of gene sets is context specific: we found that a subset of B-cell markers was conserved across tissues, while secondary markers varied from tissue to tissue. One of the strengths of FECs is that the generalizability and extensibility of a gene set can be probed with one look at the ROC curve. Either the gene set works perfectly well in the new context (AUROC = 1), or performance is suboptimal and FECs suggest how the gene set can be reorganized in the new context.

The omnipresence of FECs is compatible with the discrete organization of genes in gene sets (such as GO sets, MSigDB signatures or marker sets) and reminiscent of the polygenic model, where disease risk is distributed over a larger set of genomic loci (Golan *et al.*, 2014; Khera *et al.*, 2018; Lewis and Vassos, 2020). However, these discrete sets are usually observed in one biological (e.g. a given tissue in the marker space) or technological (PPI, co-expression data) context. Our analysis of marker generalizability across tissues suggests that, integrated across enough contexts, the degree of functionality of genes may start to appear continuous, consistent with the omnigenic model, which posits that all expressed genes are likely to contribute a disease due to the interconnections of regulatory networks (Boyle *et al.*, 2017).

Despite their rich history in genomic assessments, AUROCs are often deemed unintuitive in the presence of extreme class imbalance (Megahed *et al.*, 2021; Saito and Rehmsmeier, 2015). For example, in ROC curves extracted from the literature, positives often represented around 1% of genes (Supplementary Appendix S1). Because ROC curves set positives and negatives on the same scale, a segment of slope 10 would contribute highly to the AUROC but, within the segment, negatives would actually outnumber positives 10:1. This led to a more particular focus on the evaluation of top predictions [ROC50 (Qiu and Noble, 2008), partial AUCs (McClish, 1989; Walter, 2005) and precision-recall curves (Altman and Krzywinski, 2021; Saito and Rehmsmeier, 2015)]. On the other hand, since the shape of the ROC curve is independent of class imbalance, they facilitate global visual interpretations, e.g. local class equivalence is clearly visible as a straight line, along with other striking and interpretable patterns (Supplementary Appendix S2). Strictly speaking, FECs could also be extracted from the precision-recall curve, but they become curves in precision-recall space (see e.g. Davis and Goadrich, 2006), with highly unintuitive curvatures that depend on class imbalance (Lopes *et al.*, 2014). The visual interpretability of ROC curves was previously noted by Janssens and Martens, who attributed the occurrence of ‘angles’ to the presence of a dominant binary predictor (Janssens and Martens, 2020). In this study, we find that that ‘angles’ are widespread in genomic data because of the presence of straight lines, suggesting an underlying modular organization of the data.

Our sampling of published ROC curves suggests that FECs tend to be present regardless of the data type and prediction method used. Our focus on large aggregate databases (PPI, co-expression, drug–target interactions) confirms the presence of widespread modular structure in each data type. However, recent algorithms combine increasingly broad data resources (Gligorijević*et al.*, 2021; Kulmanov and Hoehndorf, 2020; Rifaioglu *et al.*, 2018; You *et al.*, 2021), which may smooth out the strong modularity suggested by each data type alone, thus reducing the frequency of FECs. Another interesting avenue is the integration of high-throughput functional assessments generated from CRISPR screens (Bock *et al.*, 2022), in particular the elucidation of gene function at the single-cell resolution (Replogle *et al.*, 2022), to probe the generalizability of functional modules across cell types, tissues and conditions.

In summary, FECs define a formal framework to visualize and probe the context-specificity of functional gene sets. They are simple to visualize and extract, providing a novel way to summarize complex data. They are widely applicable, as ROC curves are frequently used in genomic assessments, paving the way for comparative and meta-analytic studies. Applied across a range of contexts, they provide a first step toward teasing out shared and context-specific gene set components.

## Supplementary Material

btac692_Supplementary_Data

## Data Availability

The datasets were derived from sources in the public domain: co-expression data were obtained from the CoCoCoNet website (http://labshare.cshl.edu/shares/gillislab/resource/CoCoCoNet, 20 April 2021, date last accessed), protein-protein interaction data from BIOGRID (https://downloads.thebiogrid.org/Download/BioGRID, version 4.4.197), drug-target interaction from STITCH (http://stitch.embl.de/download, version 5.0), domain information from UniprotKB (https://rest.uniprot.org/uniprotkb, 12 August 2022, date last accessed), gene ontology information from the gene ontology website (http://current.geneontology.org/ontology/go-basic.obo, 19 December 2018, date last accessed) and the JAX website (http://www.informatics.jax.org/downloads/reports/gene_association.mgi.gz, 12 December 2018, date last accessed), data from the Tabula Muris consortium from FigShare (https://figshare.com/articles/dataset/Single-cell_RNA-seq_data_from_microfluidic_emulsion_v2_/5968960/3; https://figshare.com/articles/dataset/Single-cell_RNA-seq_data_from_Smart-seq2_sequencing_of_FACS_sorted_cells_v2_/5829687/8).
