# Supplementary material for: Defining the extent of gene function using ROC curvature
Source: Bioinformatics. 2022 Oct 22;38(24):5390–7. doi: 10.1093/bioinformatics/btac692 (PMC9750128; doi:10.1093/bioinformatics/btac692)
Supplement: btac692_Supplementary_Data [file btac692_supplementary_data.pdf]

Supplementary Information

Defining the extent of gene function using ROC curvature

Stephan Fischer[1] and Jesse Gillis[1,2,*]

[1]Cold Spring Harbor Laboratory, Stanley Institute for Cognitive Genomics, Cold Spring Harbor, NY 11724, USA

[2]Cold Spring Harbor Laboratory, Watson School of Biological Sciences, Cold Spring Harbor, NY, USA

*Correspondence: jgillis@cshl.edu

Figure S1

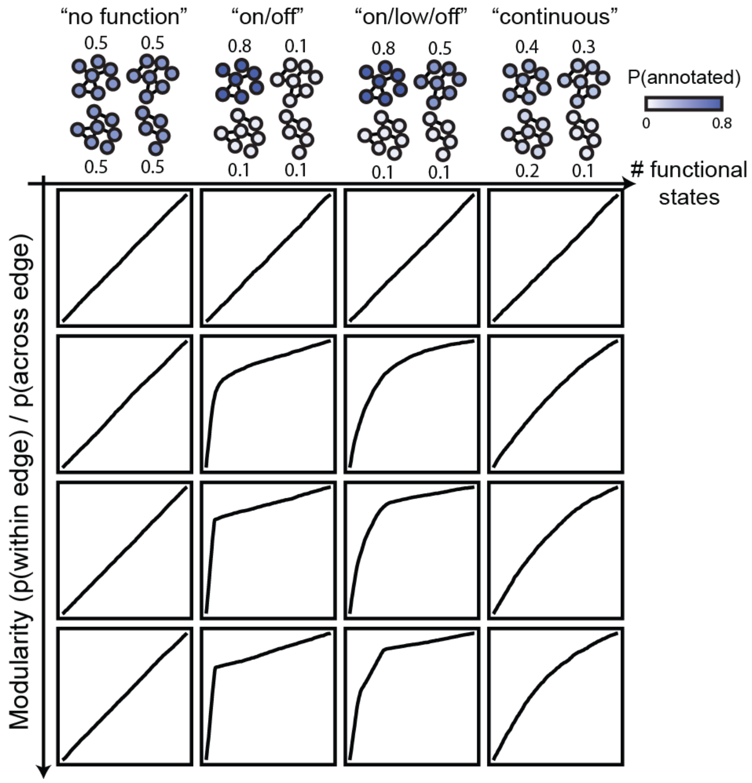

Fig. S1. Straight lines in ROC curves are associated with modular structure in network-based simulations. In these simulations, we consider 4 communities of 2,500 genes each. Each gene has a probability of being annotated according to an initial assessment, and the connection between genes follows a Stochastic Block Model structure, where the observed modularity depends on the ratio of forming edges within communities and across communities (which can be seen as a proxy of noise in the observed data). Straight lines reveal the number of functional states at low levels of noise (high observed modularities).

Figure S2

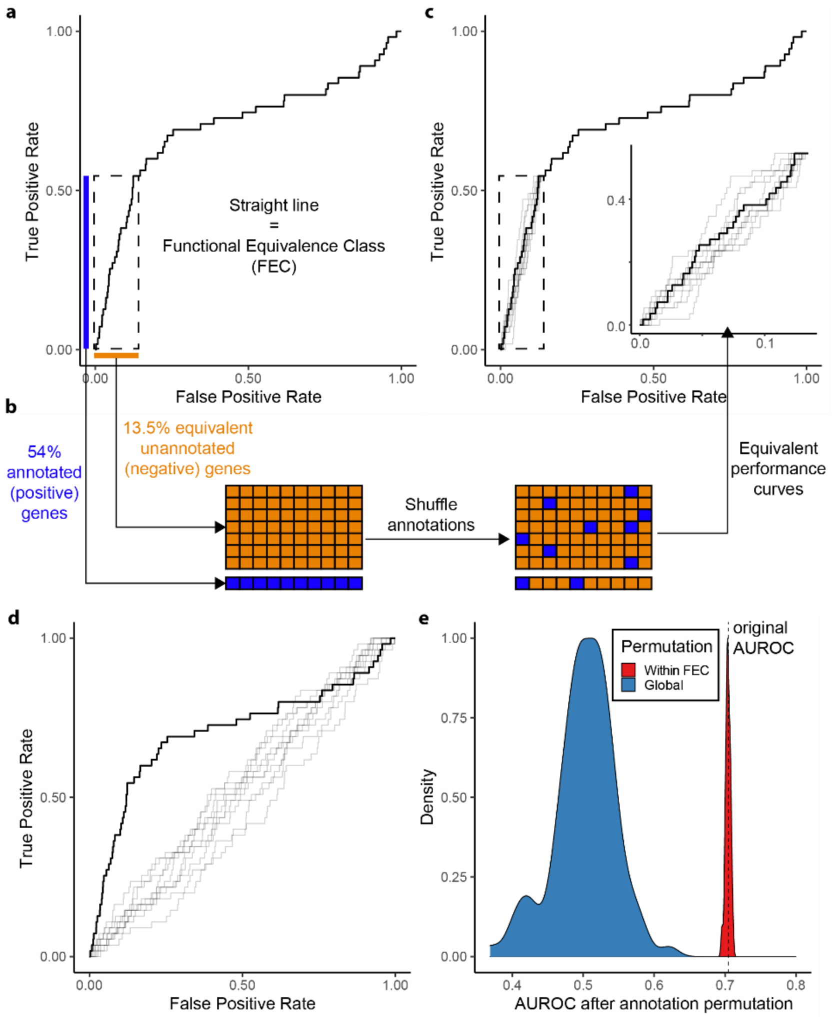

Fig. S2. Straight lines in ROC space are Functional Equivalence Classes (FECs). a Example of ROC curve obtained from a function prediction task. The initial part of the ROC curve is a FEC, a straight line containing a mix of 54% previously annotated genes and 13.5% previously unannotated genes. **b** The presence of a straight line indicates that the classifier sees positives and negatives as functionally equivalent, as if they originated from a single class. Formally, the presence of a FEC can be tested by shuffling annotations. **c** Under local annotation permutation (within the FEC), the ROC curve is essentially unchanged. The thick black line shows the original ROC curve, thin gray lines show 10 simulations of annotation permutation. The inset shows a zoom of the FEC. **d** Under global annotation permutation, the ROC curve follows the diagonal. Same color code as **c**. **e** Annotation permutations within FECs leave the AUROC essentially unchanged, while global permutations lead to a 0.5 AUROC.

Figure S3

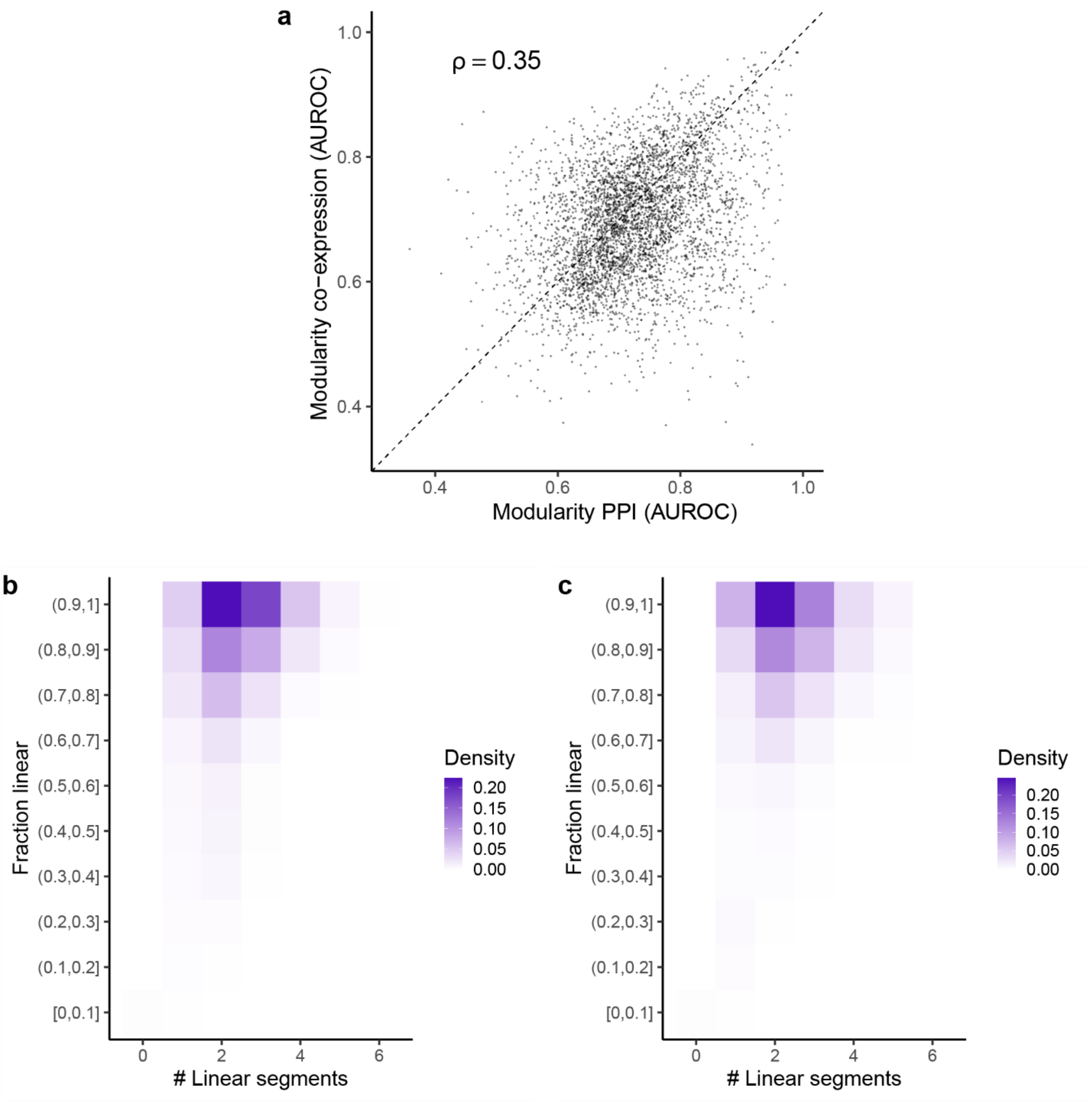

Fig. S3. ROC curves extracted from PPI and co-expression networks have correlated performance and similar shapes. a Correlation between modularity (EGAD AUROC) of GO terms in the PPI and co-expression networks. **b,c** Shape characterization of ROC curves extracted from co-expression (**b**) and PPI (**c**) networks.

Figure S4

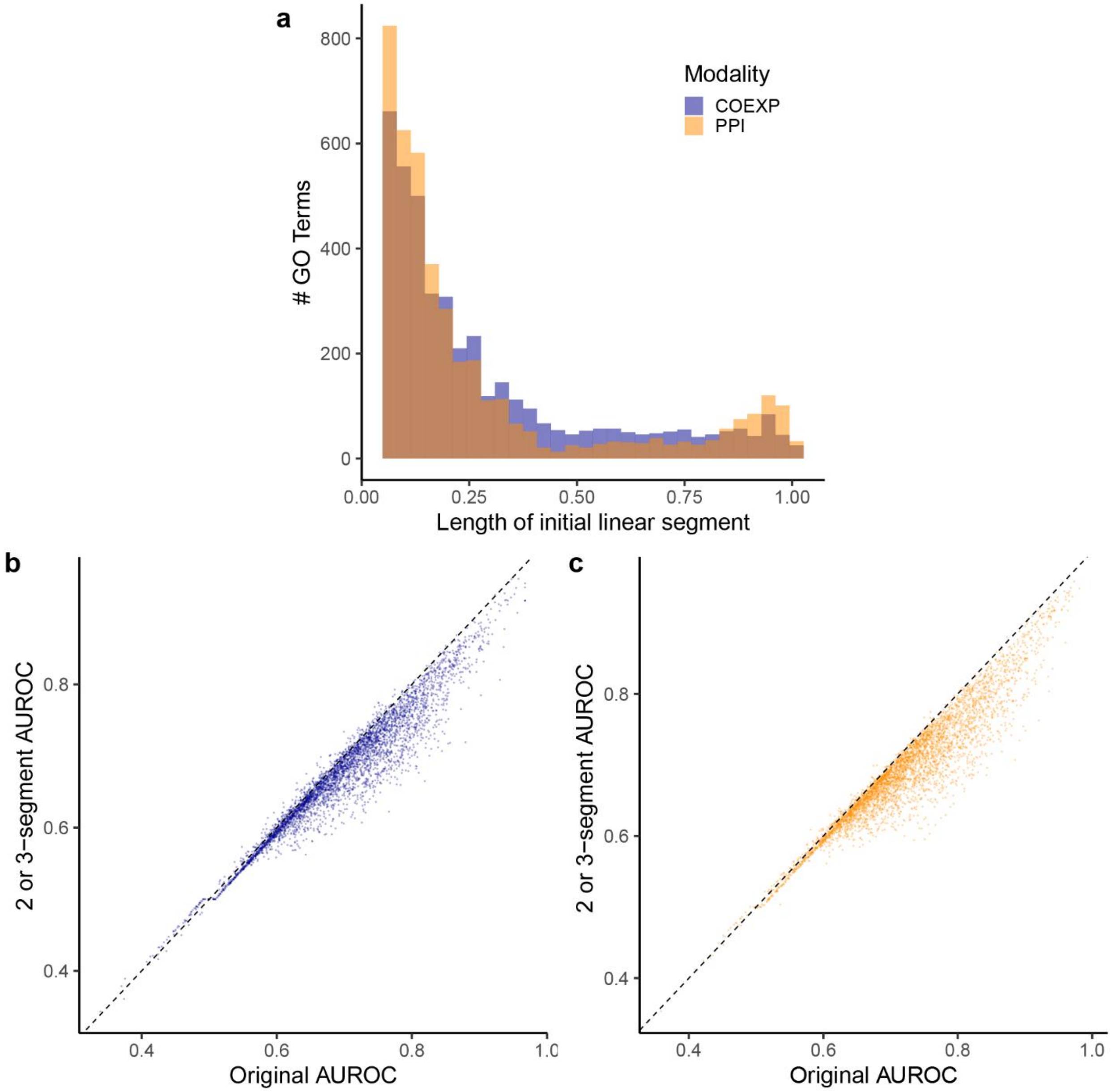

Fig. S4. Properties of FECs computed from co-expression and PPI data. a Distribution of length of the initial FECs, as measured by the fraction of the x-axis (FPR axis) spanned by the FEC.. **b,c** AUROC of 2 or 3-segment approximation of ROC curves against AUROC of original curve for co-expression data (**b**) and PPI data (**c**).

Figure S5

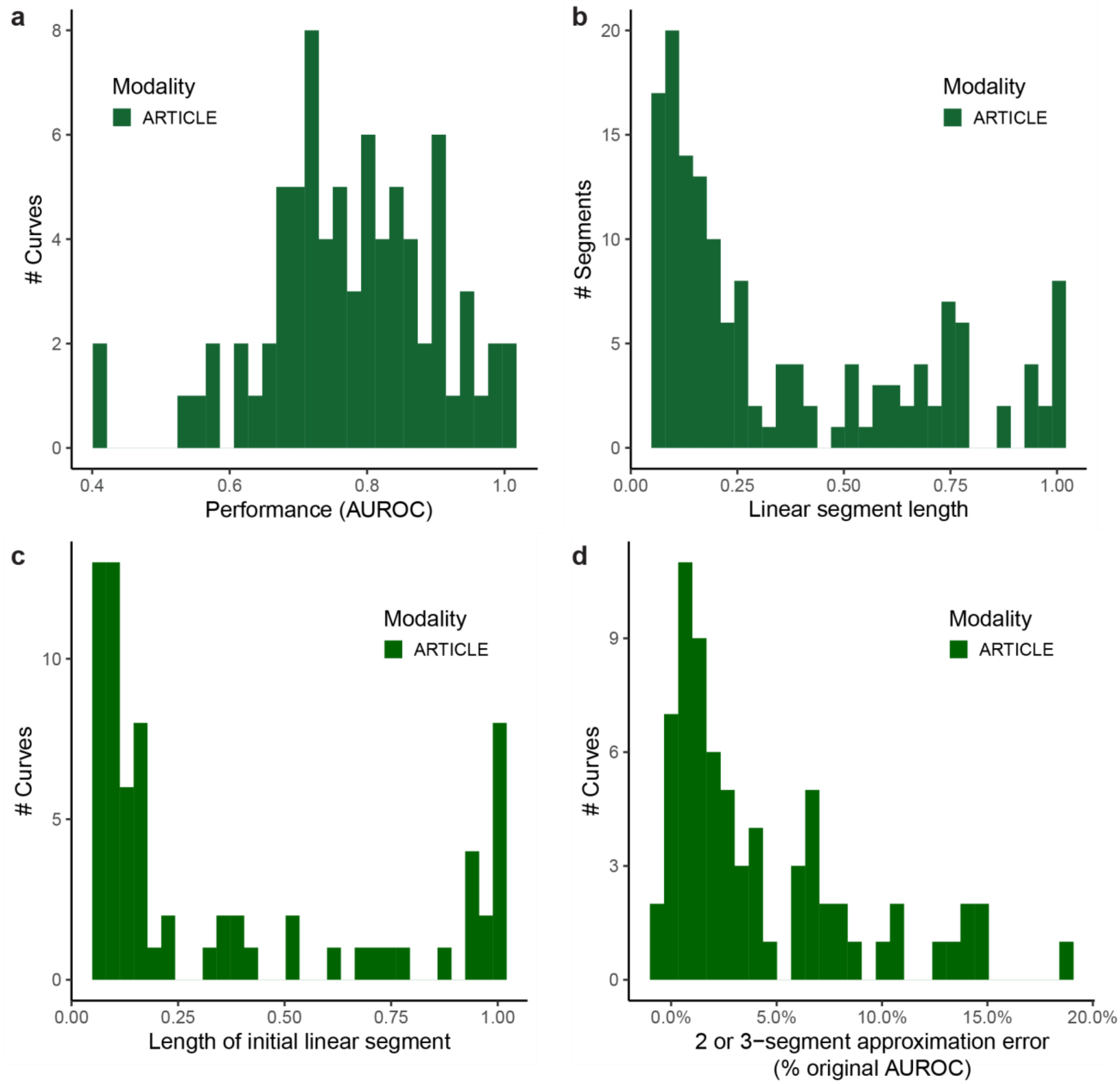

Fig. S5. Properties of FECs extracted from the literature. a Estimated performance across the 77 extracted curves. **b** Distribution of FEC lengths. **c** Distribution of length of the initial FECs, as measured by the fraction of the x-axis (FPR axis) spanned by the FEC. **d** Distribution of approximation error on the AUROC when swapping ROC curves by their 2 or 3-segment approximation. A low approximation error suggests that performance is driven by the presence of 2 or 3 discrete modules in the data.

Figure S6

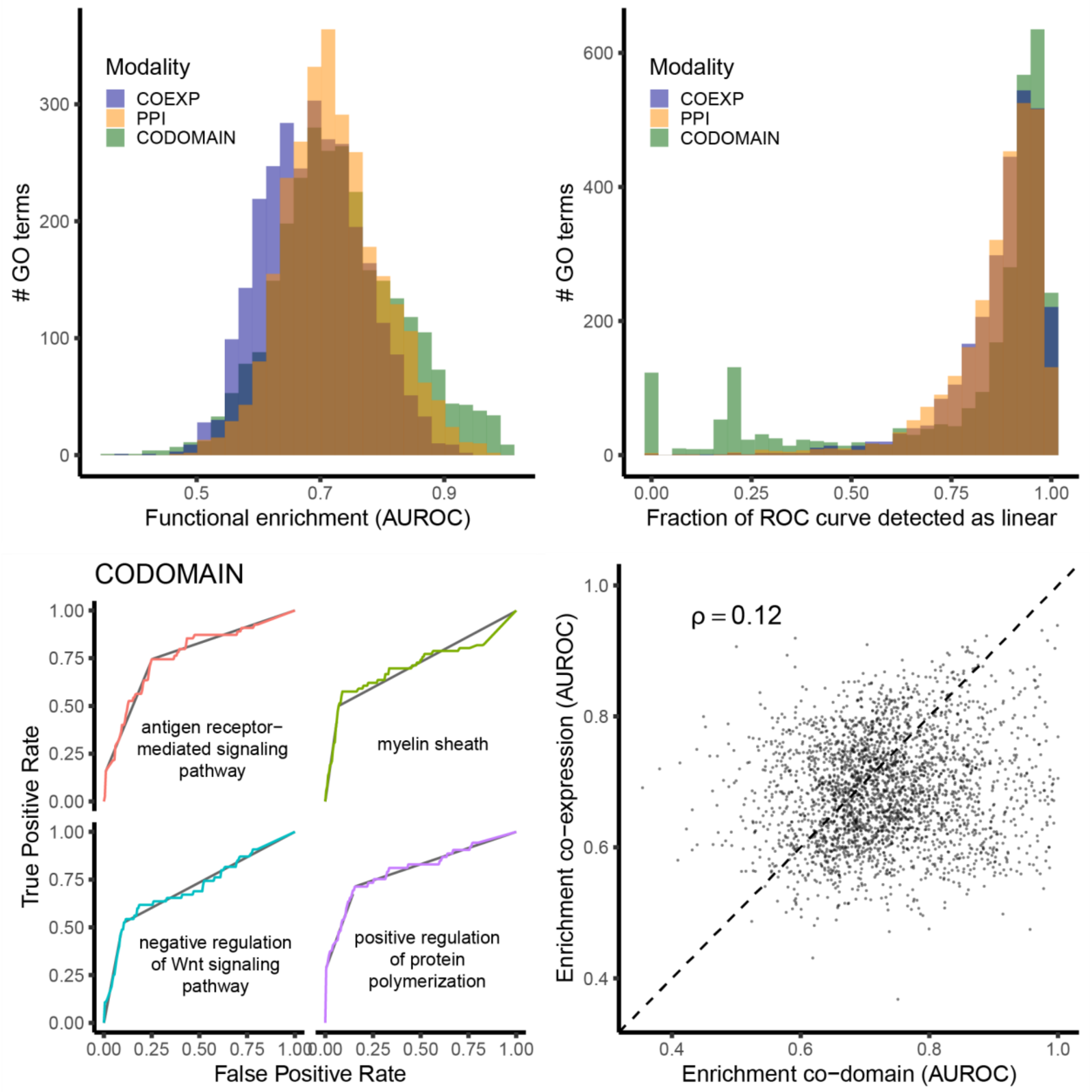

Fig. S6. ROC curves for protein function prediction from co-domain information display qualitatively similar FEC structure as co-expression and PPI-based predictions. a Average protein function prediction performance based on the 3 data types considered in the manuscript. **b** Fraction of ROC curve detected as linear. **c** Example of ROC curves derived from co-domain based predictions. **d** Scatter plot of co-domain against co-expression performance (each dot represents a GO term). Despite similar overall FEC structure, the performance of the two modalities is only weakly correlated (Spearman correlation).

Figure S7

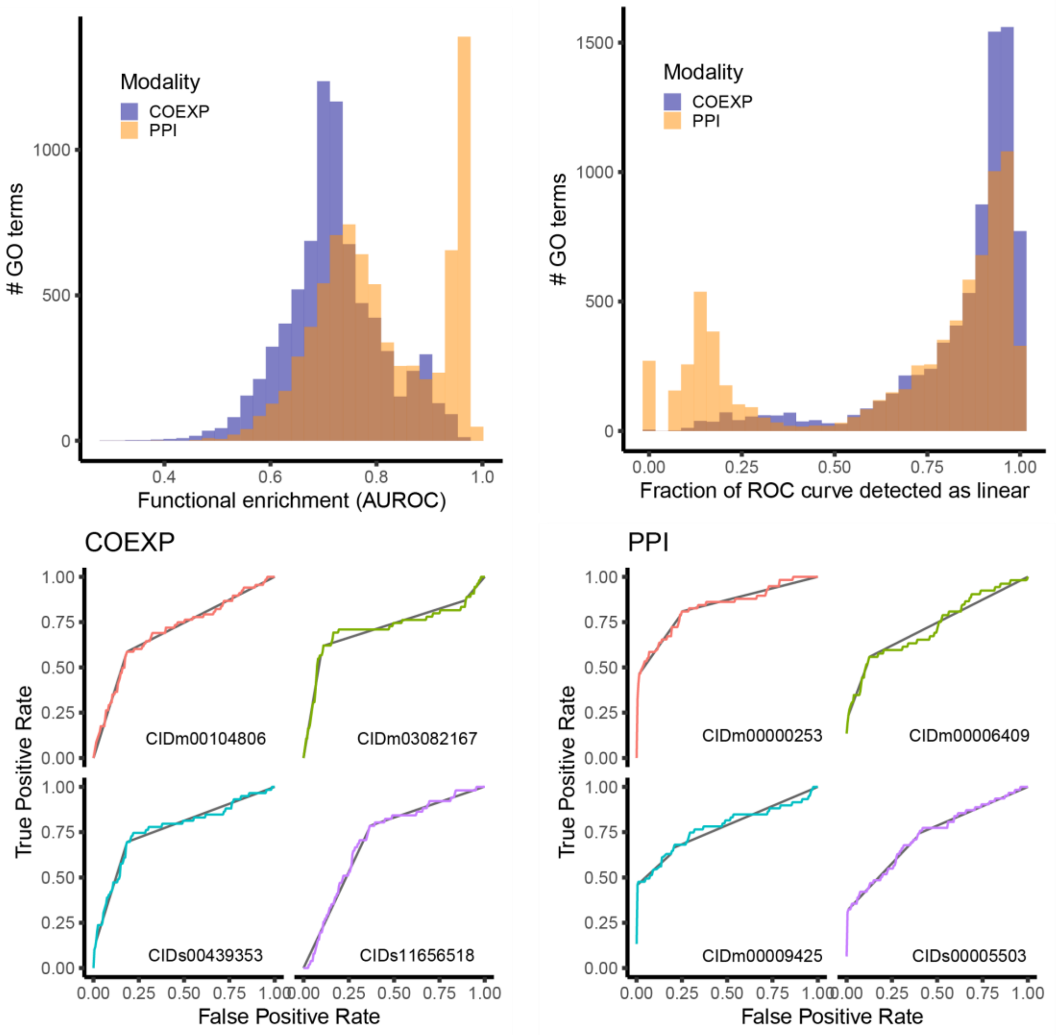

Fig. S7. FEC are equally pervasive in drug-target prediction tasks. a Performance at predicting drug-target interactions from co-expression and PPI data across various potential targets from the STITCH database. **b** Fraction of ROC curve detected as linear. **c**,**d** Examples of ROC curves derived from drug-target interaction predictions. Each facet represents a different drug.

Figure S8

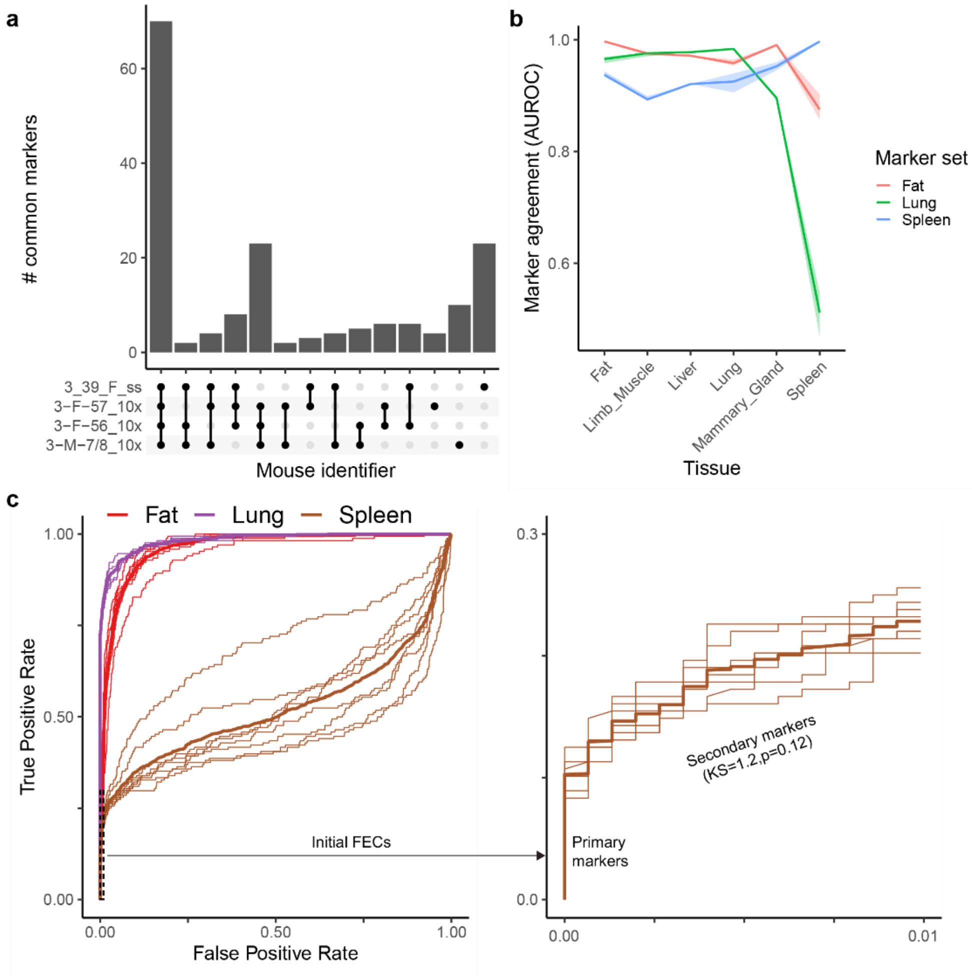

Fig. S8. Secondary markers generalize across individuals but not across tissues. a Upset plot showing the overlap across individuals of lung secondary markers. **b** Extraction of the initial FECs representing spleen-specific markers. The ROC curves represent the performance of lung consensus markers at predicting tissue-specific differentially expressed genes, thin lines show the performance for each individual in the dataset, thick lines show the average ROC curve across individuals. **c** Performance across tissues of the 3 marker sets investigated in the article.

Figure S9

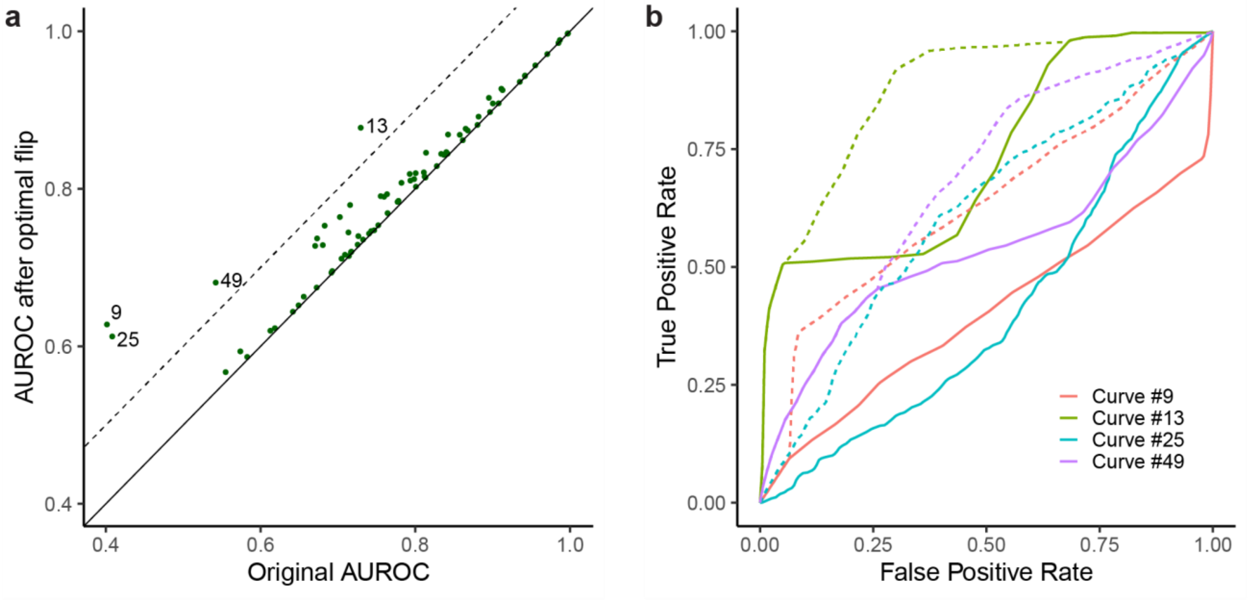

Fig. S9. Flips in ROC curves identify sections where predictions can be inverted. a Scatter plot showing optimal AUROC increase obtained by flipping a single ROC segment. The dashed line corresponds to a 0.1 AUROC increase. **b** ROC curves of the 4 curves with AUROC increase > 0.1 highlighted in **a**. Solid lines show the original ROC curve, dashed lines show the ROC curve after flipping the optimal ROC segment. In prediction space, these flips correspond to inverting the order of predictions for the genes contained in the ROC segment.

Appendix S1: List of articles and figures selected for ROC curve extraction

In the manuscript, we extracted 77 ROC curves from 50 research articles. First, we downloaded all research articles containing ROC curves in genomics-related Subject Areas from the PLoS One journal for one calendar year (2013-2014), leading to a selection of 35 articles (Table 1). We completed our selection with 15 manually selected high-profile publications (Table 2).

PUBMED ID	Figure panel	Positives	Negatives	Reference
**24551058**	Figure S2C	~100	~10000	(3)
**23741529**	Figure S1A	6000	Effectively genome-wide choose 2 (since random)	(5)
**24682035**	Figure 4B	575	Genome-wide	(2)
**24743548**	Figure 2A	190	2600	(1)
**24621610**	Figure S1A	50	Effectively genome-wide (~6000)	(4)
**24194887**	Figure 4	100	Genome-wide 6000	(6)
**23977285**	Figure 2	250	~6000 (genome-wide)	(7)
**24498199**	Figure 5	2 or more	~20000	(8)
**23922946**	Figure 10A	172	33	(9)
**24392133**	Figure 4	260	~21K	(10)
**24736605**	Figure 7	24+	10K?	(11)
**24675610**	Figure S6	2000	8244	(12)
**24349230**	Figure 5b	1485	14032	(13)
**24586446**	Figure 3C	3000	17K?	(14)
**24586611**	Figure 1			(15)
**23894279**	Figure 3	93	7000	(16)
**24260261**	Figure 1	3638	Random 3638	(17)
**24194902**	Figure 2	~2K	~10K	(18)
**24236095**	Figure 4E	163	163	(19)
**24098743**	Figure 3	300	Random 1000	(20)
**24489849**	Figure 5			(21)
**24699297**	Figure 7C			(22)
**24349035**	Figure 1	259	259 choose 2	(23)
**24454733**	Figure 2	6000	6000	(24)
**23874989**	Figure 3	270	562*100?	(25)
**24194827**	Figure 1			(26)
**24376739**	Figure 3	79	119070	(27)
**24391954**	Figure 7	35	86	(28)
**24349449**	Figure 5			(29)
**24475169**	Figure 3	93	93	(30)
**24019945**	Figure 4	109	1700	(31)
**23950912**	Figure 6A			(32)
**24069417**	Figure 6	4753	17793	(33)
**23675414**	Figure 10			(34)
**23690949**	Figure S1			(35)

Table S1. Publications containing genomics-related ROC curves from the PLoS One journal.

PUBMED ID	Figure panel	Positives	Negatives	Reference
**18371930**	Figure 3	783		(36)
**24156763**	Figure S9	200	~200	(37)
**16685651**	Figure 4C	409		(38)
**22681890**	Figure S1B			(39)
**23545499**	Figure 5B	~100		(40)

**18724933**	Figure S2C		(41)
**23932120**	Figure 2A		(42)
**20813266**	Figure S3A	125	(43)
**16680138**	Figure 3B	627	(44)
**24813450**	Figure 3C	200	(45)
**20118918**	Figure 2F	~30	(46)
**24114784**	Figure 1B	150	(47)
**22344438**	Figure 3C	253	(48)
**19690572**	Figure 1A	834	(49)
**15998909**	Figure 3A		(50)

Table S2. Selection of high-profile publications containing ROC curves.

Appendix S2 : discussion of interpretable ROC patterns beyond straight segments

As we discuss in the manuscript, ROC curves extracted from the literature have several visual characteristics that inform about the prediction task and data, such as the AUROC, the ROC50 metric, or the presence of straight segments. We show that these characteristics are surprisingly similar across a large body of literature, prediction tasks and data sources (e.g., PPI or co-expression data)
However, there is another striking pattern that we found exclusively in ROC curves extracted from the published literature: for 4/71 (6%) curves, flipping a segment of the curve significantly increased the AUROC performance (Fig. S9). “Flippable segments” were notably absent in ROC curves computed from co-expression and PPI data, suggesting that they are likely related to extreme data distributions or methodological issues. In some instances, optimal performance was achieved by flipping the whole curve (i.e., completely inverting predictions); in others, the curve was locally S-shaped and the optimal flip only contained around 50% of the curve (Fig. S9). The latter case suggests that the best predictions are located in the initial and final part of the S, which would typically arise by mistakenly treating a two-sided assessment (where both highly positive and highly negative predictions should be considered “high”) as one-sided. This scenario is likely to arise, e.g., in the context of a benchmark, where methods are applied with default or suboptimal parameters.
Similar to straight segments, the interpretation of flipped segments becomes more intuitive when ROC curves are interpreted as 2-dimensional ranked lists (Fig. 2), which better convey the understanding that all or part of the predictions are reversed. As a result, we argue that recurrent or suprising ROC patterns usually can - and should - be carefully examined and interpretated.

References

1. Cui S, Youn E, Lee J, Maas SJ. An improved systematic approach to predicting transcription factor target genes using support vector machine. PloS one. 2014;9(4):e94519. doi: 10.1371/journal.pone.0094519. PubMed PMID: 24743548; PubMed Central PMCID: PMC3990533.
2. Ersahin T, Carkacioglu L, Can T, Konu O, Atalay V, Cetin-Atalay R. Identification of novel reference genes based on MeSH categories. PloS one. 2014;9(3):e93341. doi: 10.1371/journal.pone.0093341. PubMed PMID: 24682035; PubMed Central PMCID: PMC3969360.
3. Guo X, Zhang Y, Hu W, Tan H, Wang X. Inferring nonlinear gene regulatory networks from gene expression data based on distance correlation. PloS one. 2014;9(2):e87446. doi: 10.1371/journal.pone.0087446. PubMed PMID: 24551058; PubMed Central PMCID: PMC3925093.
4. Liao B, Li Y, Jiang Y, Cai L. Using multi-instance hierarchical clustering learning system to predict yeast gene function. PloS one. 2014;9(3):e90962. doi: 10.1371/journal.pone.0090962. PubMed PMID: 24621610; PubMed Central PMCID: PMC3951281.
5. Wu X, Pang E, Lin K, Pei ZM. Improving the measurement of semantic similarity between gene ontology terms and gene products: insights from an edge- and IC-based hybrid method. PloS one. 2013;8(5):e66745. doi: 10.1371/journal.pone.0066745. PubMed PMID: 23741529; PubMed Central PMCID: PMC3669204.

6. Wang P, Lai WF, Li MJ, Xu F, Yalamanchili HK, Lovell-Badge R, et al. Inference of gene-phenotype associations via protein-protein interaction and orthology. PloS one. 2013;8(10):e77478. doi: 10.1371/journal.pone.0077478. PubMed PMID: 24194887; PubMed Central PMCID: PMC3806783.
7. Wei W, Ning LW, Ye YN, Guo FB. Geptop: a gene essentiality prediction tool for sequenced bacterial genomes based on orthology and phylogeny. PloS one. 2013;8(8):e72343. doi: 10.1371/journal.pone.0072343. PubMed PMID: 23977285; PubMed Central PMCID: PMC3744497.
8. Yang X, Gao L, Guo X, Shi X, Wu H, Song F, et al. A network based method for analysis of lncRNA-disease associations and prediction of lncRNAs implicated in diseases. PloS one. 2014;9(1):e87797. doi: 10.1371/journal.pone.0087797. PubMed PMID: 24498199; PubMed Central PMCID: PMC3909255.
9. Mendoza MR, da Fonseca GC, Loss-Morais G, Alves R, Margis R, Bazzan AL. RFMirTarget: predicting human microRNA target genes with a random forest classifier. PloS one. 2013;8(7):e70153. doi: 10.1371/journal.pone.0070153. PubMed PMID: 23922946; PubMed Central PMCID: PMC3724815.
10. Liu MX, Chen X, Chen G, Cui QH, Yan GY. A computational framework to infer human disease-associated long noncoding RNAs. PloS one. 2014;9(1):e84408. doi: 10.1371/journal.pone.0084408. PubMed PMID: 24392133; PubMed Central PMCID: PMC3879311.
11. Rezaeian I, Rueda L. CMT: A Constrained Multi-Level Thresholding Approach for ChIP-Seq Data Analysis. PloS one. 2014;9(4):e93873. doi: 10.1371/journal.pone.0093873. PubMed PMID: 24736605; PubMed Central PMCID: PMC3988018.
12. Li L, Cui X, Yu S, Zhang Y, Luo Z, Yang H, et al. PSSP-RFE: accurate prediction of protein structural class by recursive feature extraction from PSI-BLAST profile, physical-chemical property and functional annotations. PloS one. 2014;9(3):e92863. doi: 10.1371/journal.pone.0092863. PubMed PMID: 24675610; PubMed Central PMCID: PMC3968047.
13. Eichner J, Topf F, Drager A, Wrzodek C, Wanke D, Zell A. TFpredict and SABINE: sequence-based prediction of structural and functional characteristics of transcription factors. PloS one. 2013;8(12):e82238. doi: 10.1371/journal.pone.0082238. PubMed PMID: 24349230; PubMed Central PMCID: PMC3861411.
14. Krepelova A, Neri F, Maldotti M, Rapelli S, Oliviero S. Myc and max genome-wide binding sites analysis links the Myc regulatory network with the polycomb and the core pluripotency networks in mouse embryonic stem cells. PloS one. 2014;9(2):e88933. doi: 10.1371/journal.pone.0088933. PubMed PMID: 24586446; PubMed Central PMCID: PMC3931652.
15. Talebzadeh M, Zare-Mirakabad F. Transcription factor binding sites prediction based on modified nucleosomes. PloS one. 2014;9(2):e89226. doi: 10.1371/journal.pone.0089226. PubMed PMID: 24586611; PubMed Central PMCID: PMC3931712.
16. Wong ES, Hardy MC, Wood D, Bailey T, King GF. SVM-based prediction of propeptide cleavage sites in spider toxins identifies toxin innovation in an Australian tarantula. PloS one. 2013;8(7):e66279. doi: 10.1371/journal.pone.0066279. PubMed PMID: 23894279; PubMed Central PMCID: PMC3718798.
17. Mei S. Probability weighted ensemble transfer learning for predicting interactions between HIV-1 and human proteins. PloS one. 2013;8(11):e79606. doi: 10.1371/journal.pone.0079606. PubMed PMID: 24260261; PubMed Central PMCID: PMC3832534.
18. Hu J, Ng PC. SIFT Indel: predictions for the functional effects of amino acid insertions/deletions in proteins. PloS one. 2013;8(10):e77940. doi: 10.1371/journal.pone.0077940. PubMed PMID: 24194902; PubMed Central PMCID: PMC3806772.
19. Blomquist TM, Crawford EL, Lovett JL, Yeo J, Stanoszek LM, Levin A, et al. Targeted RNA-sequencing with competitive multiplex-PCR amplicon libraries. PloS one. 2013;8(11):e79120. doi: 10.1371/journal.pone.0079120. PubMed PMID: 24236095; PubMed Central PMCID: PMC3827295.
20. Lofthouse EK, Wheeler PR, Beste DJ, Khatri BL, Wu H, Mendum TA, et al. Systems-based approaches to probing metabolic variation within the Mycobacterium tuberculosis complex. PloS one. 2013;8(9):e75913. doi: 10.1371/journal.pone.0075913. PubMed PMID: 24098743; PubMed Central PMCID: PMC3783153.
21. de Moraes FR, Neshich IA, Mazoni I, Yano IH, Pereira JG, Salim JA, et al. Improving predictions of protein-protein interfaces by combining amino acid-specific classifiers based on structural and physicochemical descriptors with their weighted neighbor averages. PloS one. 2014;9(1):e87107. doi: 10.1371/journal.pone.0087107. PubMed PMID: 24489849; PubMed Central PMCID: PMC3904977.

22. van Westen GJ, Gaulton A, Overington JP. Chemical, target, and bioactive properties of allosteric modulation. PLoS computational biology. 2014;10(4):e1003559. doi: 10.1371/journal.pcbi.1003559. PubMed PMID: 24699297; PubMed Central PMCID: PMC3974644.
23. Zhou H, Jakobsson E. Predicting protein-protein interaction by the mirrortree method: possibilities and limitations. PloS one. 2013;8(12):e81100. doi: 10.1371/journal.pone.0081100. PubMed PMID: 24349035; PubMed Central PMCID: PMC3862474.
24. Espinosa O, Mitsopoulos K, Hakas J, Pearl F, Zvelebil M. Deriving a mutation index of carcinogenicity using protein structure and protein interfaces. PloS one. 2014;9(1):e84598. doi: 10.1371/journal.pone.0084598. PubMed PMID: 24454733; PubMed Central PMCID: PMC3893166.
25. Sun J, Zhou M, Yang H, Deng J, Wang L, Wang Q. Inferring potential microRNA-microRNA associations based on targeting propensity and connectivity in the context of protein interaction network. PloS one. 2013;8(7):e69719. doi: 10.1371/journal.pone.0069719. PubMed PMID: 23874989; PubMed Central PMCID: PMC3713046.
26. Liu L, Zhang Z, Mei Q, Chen M. PSI: a comprehensive and integrative approach for accurate plant subcellular localization prediction. PloS one. 2013;8(10):e75826. doi: 10.1371/journal.pone.0075826. PubMed PMID: 24194827; PubMed Central PMCID: PMC3806775.
27. Ma Z, Axtell MJ. Long-range genomic enrichment, sequencing, and assembly to determine unknown sequences flanking a known microRNA. PloS one. 2013;8(12):e83721. doi: 10.1371/journal.pone.0083721. PubMed PMID: 24376739; PubMed Central PMCID: PMC3869802.
28. Yang X, Guo Y, Luo J, Pu X, Li M. Effective identification of Gram-negative bacterial type III secreted effectors using position-specific residue conservation profiles. PloS one. 2013;8(12):e84439. doi: 10.1371/journal.pone.0084439. PubMed PMID: 24391954; PubMed Central PMCID: PMC3877298.
29. Zhou Y, Liu S, Song J, Zhang Z. Structural propensities of human ubiquitination sites: accessibility, centrality and local conformation. PloS one. 2013;8(12):e83167. doi: 10.1371/journal.pone.0083167. PubMed PMID: 24349449; PubMed Central PMCID: PMC3859641.
30. Lou W, Wang X, Chen F, Chen Y, Jiang B, Zhang H. Sequence based prediction of DNA-binding proteins based on hybrid feature selection using random forest and Gaussian naive Bayes. PloS one. 2014;9(1):e86703. doi: 10.1371/journal.pone.0086703. PubMed PMID: 24475169; PubMed Central PMCID: PMC3901691.
31. Chen X, Qiu JD, Shi SP, Suo SB, Liang RP. Systematic analysis and prediction of pupylation sites in prokaryotic proteins. PloS one. 2013;8(9):e74002. doi: 10.1371/journal.pone.0074002. PubMed PMID: 24019945; PubMed Central PMCID: PMC3760804.
32. Xuan P, Han K, Guo M, Guo Y, Li J, Ding J, et al. Prediction of microRNAs associated with human diseases based on weighted k most similar neighbors. PloS one. 2013;8(8):e70204. doi: 10.1371/journal.pone.0070204. PubMed PMID: 23950912; PubMed Central PMCID: PMC3738541.
33. Dogan T, Karacali B. Automatic identification of highly conserved family regions and relationships in genome wide datasets including remote protein sequences. PloS one. 2013;8(9):e75458. doi: 10.1371/journal.pone.0075458. PubMed PMID: 24069417; PubMed Central PMCID: PMC3771926.
34. Nilmeier JP, Kirshner DA, Wong SE, Lightstone FC. Rapid catalytic template searching as an enzyme function prediction procedure. PloS one. 2013;8(5):e62535. doi: 10.1371/journal.pone.0062535. PubMed PMID: 23675414; PubMed Central PMCID: PMC3651201.
35. Kundu K, Costa F, Huber M, Reth M, Backofen R. Semi-supervised prediction of SH2-peptide interactions from imbalanced high-throughput data. PloS one. 2013;8(5):e62732. doi: 10.1371/journal.pone.0062732. PubMed PMID: 23690949; PubMed Central PMCID: PMC3656881.
36. Kohler S, Bauer S, Horn D, Robinson PN. Walking the interactome for prioritization of candidate disease genes. American journal of human genetics. 2008;82(4):949-58. doi: 10.1016/j.ajhg.2008.02.013. PubMed PMID: 18371930; PubMed Central PMCID: PMC2427257.
37. Taher L, Smith RP, Kim MJ, Ahituv N, Ovcharenko I. Sequence signatures extracted from proximal promoters can be used to predict distal enhancers. Genome biology. 2013;14(10):R117. doi: 10.1186/gb-2013-14-10-r117. PubMed PMID: 24156763; PubMed Central PMCID: PMC3983659.
38. Franke L, van Bakel H, Fokkens L, de Jong ED, Egmont-Petersen M, Wijmenga C. Reconstruction of a functional human gene network, with an application for prioritizing positional candidate genes. American journal of human genetics. 2006;78(6):1011-25. doi: 10.1086/504300. PubMed PMID: 16685651; PubMed Central PMCID: PMC1474084.

39. Ryan CJ, Roguev A, Patrick K, Xu J, Jahari H, Tong Z, et al. Hierarchical modularity and the evolution of genetic interactomes across species. Molecular cell. 2012;46(5):691-704. doi: 10.1016/j.molcel.2012.05.028. PubMed PMID: 22681890; PubMed Central PMCID: PMC3380636.
40. Tinti M, Kiemer L, Costa S, Miller ML, Sacco F, Olsen JV, et al. The SH2 domain interaction landscape. Cell reports. 2013;3(4):1293-305. doi: 10.1016/j.celrep.2013.03.001. PubMed PMID: 23545499.
41. Alekseyenko AA, Peng S, Larschan E, Gorchakov AA, Lee OK, Kharchenko P, et al. A sequence motif within chromatin entry sites directs MSL establishment on the Drosophila X chromosome. Cell. 2008;134(4):599-609. doi: 10.1016/j.cell.2008.06.033. PubMed PMID: 18724933; PubMed Central PMCID: PMC2613042.
42. Braberg H, Jin H, Moehle EA, Chan YA, Wang S, Shales M, et al. From structure to systems: high-resolution, quantitative genetic analysis of RNA polymerase II. Cell. 2013;154(4):775-88. doi: 10.1016/j.cell.2013.07.033. PubMed PMID: 23932120; PubMed Central PMCID: PMC3932829.
43. Ohta S, Bukowski-Wills JC, Sanchez-Pulido L, Alves Fde L, Wood L, Chen ZA, et al. The protein composition of mitotic chromosomes determined using multiclassifier combinatorial proteomics. Cell. 2010;142(5):810-21. doi: 10.1016/j.cell.2010.07.047. PubMed PMID: 20813266; PubMed Central PMCID: PMC2982257.
44. Aerts S, Lambrechts D, Maity S, Van Loo P, Coessens B, De Smet F, et al. Gene prioritization through genomic data fusion. Nature biotechnology. 2006;24(5):537-44. doi: 10.1038/nbt1203. PubMed PMID: 16680138.
45. Cho A, Shin J, Hwang S, Kim C, Shim H, Kim H, et al. WormNet v3: a network-assisted hypothesis-generating server for Caenorhabditis elegans. Nucleic acids research. 2014. doi: 10.1093/nar/gku367. PubMed PMID: 24813450.
46. Lee I, Ambaru B, Thakkar P, Marcotte EM, Rhee SY. Rational association of genes with traits using a genome-scale gene network for Arabidopsis thaliana. Nature biotechnology. 2010;28(2):149-56. doi: 10.1038/nbt.1603. PubMed PMID: 20118918; PubMed Central PMCID: PMC2857375.
47. Kwon Y, Vinayagam A, Sun X, Dephoure N, Gygi SP, Hong P, et al. The Hippo signaling pathway interactome. Science. 2013;342(6159):737-40. doi: 10.1126/science.1243971. PubMed PMID: 24114784; PubMed Central PMCID: PMC3951131.
48. MacArthur DG, Balasubramanian S, Frankish A, Huang N, Morris J, Walter K, et al. A systematic survey of loss-of-function variants in human protein-coding genes. Science. 2012;335(6070):823-8. doi: 10.1126/science.1215040. PubMed PMID: 22344438; PubMed Central PMCID: PMC3299548.
49. Li J, Zimmerman LJ, Park BH, Tabb DL, Liebler DC, Zhang B. Network-assisted protein identification and data interpretation in shotgun proteomics. Molecular systems biology. 2009;5:303. doi: 10.1038/msb.2009.54. PubMed PMID: 19690572; PubMed Central PMCID: PMC2736651.
50. Lu LJ, Xia Y, Paccanaro A, Yu H, Gerstein M. Assessing the limits of genomic data integration for predicting protein networks. Genome research. 2005;15(7):945-53. doi: 10.1101/gr.3610305. PubMed PMID: 15998909; PubMed Central PMCID: PMC1172038.
